# Effectiveness of pregnant women’s active participation in their antenatal care for the control of malaria and anaemia in pregnancy in Ghana: a cluster randomized controlled trial

**DOI:** 10.1186/s12936-018-2387-1

**Published:** 2018-06-19

**Authors:** Gifty Dufie Ampofo, Harry Tagbor, Imelda Bates

**Affiliations:** 1 University of Health and Allied Sciences, PMB 31, Ho, Ghana; 2 Liverpool School of Tropical Medicine, Liverpool, UK

**Keywords:** Antenatal care, ANC, Malaria and anaemia in pregnancy, Active participation in antenatal care, Rapid diagnostic test, Haemoglobin colour scale, Ghana

## Abstract

**Background:**

The burden of malaria and anaemia in pregnancy remains high despite the availability of proven efficacious antenatal care interventions. Sub-optimal uptake of the interventions may be due to inadequate active participation of pregnant women in their antenatal care. It was hypothesized that providing opportunities for pregnant women to improve upon active participation in their antenatal care through malaria and anaemia point-of-care testing would improve adherence to ANC recommendations and interventions and lead to better pregnancy outcomes.

**Methods:**

Fourteen antenatal clinics in the Ashanti region of Ghana were randomized into intervention (pregnant women participating in their care plus current routine care) and control (current routine care) arms. Pregnant women attending the clinics for the first time were recruited and followed up until delivery. Haemoglobin levels and malaria parasitaemia were measured at baseline, 4–8 weeks after recruitment and at 36–40 weeks gestation. Birth weight and pregnancy outcomes were also recorded.

**Results:**

The overall mean age, gestational age and haemoglobin at baseline were 26.4 years, 17.3 weeks and 110 g/l, respectively, with no significant differences between groups; 10.7% had asymptomatic parasitaemia; 74.6% owned an ITN but only 48.8% slept under it the night before enrolment. The adjusted risk ratio by 8 weeks follow up and at 36–40 weeks gestation in the intervention versus the control was 0.97 (95% CI 0.78–1.22) and 0.92 (95% CI 0.63–1.34) for anaemia and 1.17 (95% CI 0.68–2.04) and 0.83 (95% CI 0.27–2.57) for parasitaemia. The adjusted risk ratio for low birth weight was 0.93 (95% CI 0.44–1.97) and for pregnancy complications (abortions, intrauterine fetal deaths and still births) was 0.77 (95% CI 0.17–3.52) in the intervention group versus controls.

**Conclusion:**

Although its potential was evident, this study found no significant beneficial effect of women participating in their malaria and haemoglobin tests on pregnancy outcomes. Exploring factors influencing health worker compliance to health intervention implementation and patient adherence to health interventions within this context will contribute in future to improving intervention effectiveness.

*Trial registration* ISRTCTN88917252

**Electronic supplementary material:**

The online version of this article (10.1186/s12936-018-2387-1) contains supplementary material, which is available to authorized users.

## Background

Malaria and anemia [hemoglobin (Hb) < 110 g/l] during pregnancy in sub-Saharan Africa remain a concern despite proven efficacious antenatal care (ANC) interventions. Recommended ANC interventions include Intermittent preventive treatment of malaria during pregnancy (IPTp) using Sulfadoxine–pyrimethamine (SP), the use of insecticide-treated bed nets (ITN) and ensuring effective case management of malaria illness and anaemia [1]. ANC coverage in Ghana has improved over the last two and a half decades with more than 95% of pregnant women attending ANC clinics at least once during their pregnancy and 87% four or more times [2]. It is expected that as pregnant women visit the clinics they receive all these efficacious ANC interventions but their pregnancy outcomes show otherwise. In Ghana, anaemia and asymptomatic malaria prevalence and their detrimental effects during pregnancy continue to be of public health concern. Almost 50% of pregnant women at term (36–40 weeks gestation) and 12–36% of women at 32 weeks gestation and above, have asymptomatic malaria parasitaemia [3–5]. Eleven percent of babies born are of low birth weight [3] and ITN use during pregnancy is only 32%. Current national statistics show that 68% of pregnant women received two or more doses of IPTp. Fifty-nine percent of them reported taking their iron supplements for 3 months or more [2]. Factors that contribute to low intervention effectiveness include inaccessibility or unavailability of ANC clinics, unavailability of resources needed for intervention implementation and lack of adherence of ANC staff and pregnant women to these interventions [6]. Health system issues such as unclear policy and guidance, stock outs, user fees, poor organization leading to poor quality of care, poor healthcare provider performance as well as factors concerning pregnant women such as poor timing of ANC clinic visits, education, age, marital status, knowledge about malaria and IPTp, socio-economic status and parity have been identified as key determinants to the uptake of IPTp and ITN during antenatal care [7].

The focus of efforts to enhance effectiveness of ANC interventions has mainly been on improving health services for better health outcomes. While this occurs mainly in the clinical settings, evidence has also shown that community-based health intervention packages involving health personnel such as community health workers and community health nurses can improve maternal and child survival [8, 9]. Encouraging patients to actively participate in their healthcare leads to better health outcomes [10, 11] through improved adherence to or uptake of health interventions [12, 13]. Encouraging patients to take part in their health care decisions is considered ‘a sign of valuing humanity and the individuality of the patient,’ a legal right of the patient as well as an international gold standard for healthcare systems’ [14]. Although this concept has been applied to some aspects of patient care including decision-making during medical consultation, patient safety and the management of some chronic diseases [15–17], little is known about its usefulness in antenatal care. Only a few studies have targeted strategies aimed at improving adherence of pregnant women to ANC interventions. Mbonye and colleagues have showed that by creating awareness about IPTp and its importance and improving health worker-pregnant woman trust, pregnant women can be influenced to improve their adherence to scheduled ANC visits, 2 doses of IPTp and health facility-based delivery [18] albeit not through active participation.

**Fig. 1:**
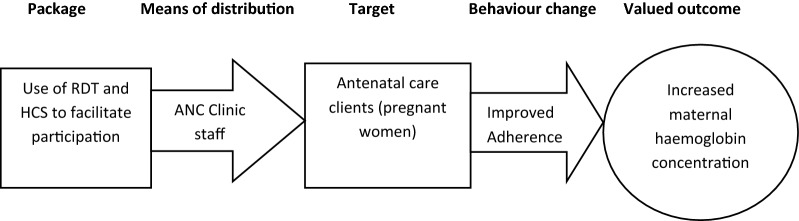
Conceptual framework for pregnant women’s active participation in their antenatal care (Adapted from Campbell and Graham [33])

This study hypothesized that when pregnant women actively participate in their ANC, this would improve their adherence and hence uptake of ANC interventions translating eventually into better health outcomes (Fig. 1). This concept was tested in this study in relation to the control of malaria and anaemia in pregnancy. Active participation in this study means allowing pregnant women to see the conduct and results of their point-of-care tests for malaria and anaemia and be involved with the interpretation of the test results so as to influence their decision to adhere to ANC treatments and recommendations given at the ANC.

## Methods

### Study area and population

**Fig. 2:**
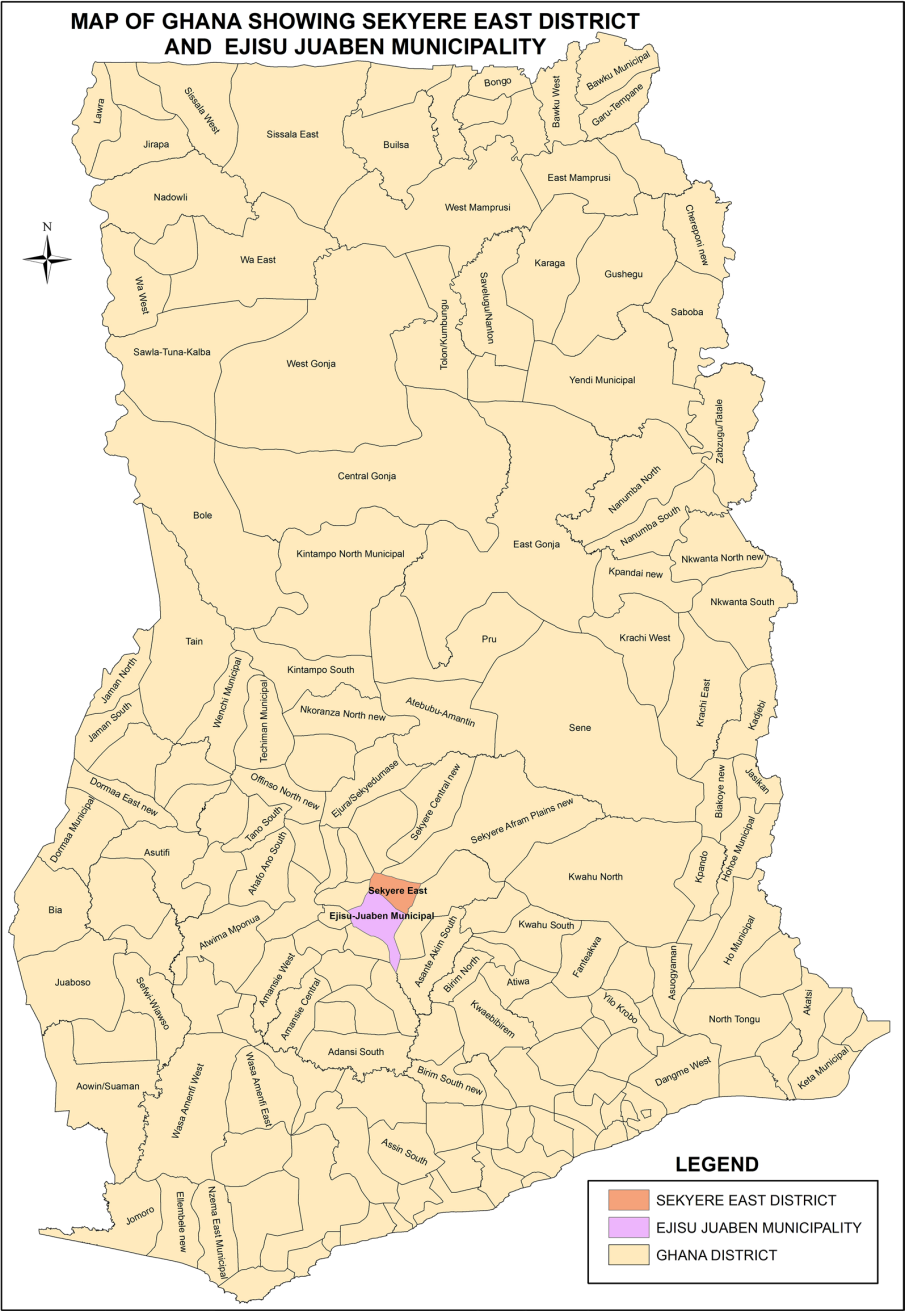
The Sekyere-East district and Ejisu-Juaben Municipality, Ashanti region, Ghana

The trial was conducted in the Ejisu-Juaben municipality and the Sekyere-East district of the Ashanti region of Ghana (Fig. 2) from September 2012 to April 2014. These two areas lie adjacent to each other in Ghana’s forest region. The Sekyere-East district is predominantly rural while the Ejisu-Juaben municipality has more semi-urban areas. There are a total of four government hospitals in both areas, which serve as referral points for smaller health centres, clinics and maternity homes offering antenatal care. In 2011, antenatal care coverage in the Ejisu-Juaben municipality was 96.1% with an average of 3.7 visits per woman [19] while that of the Sekyere-East district was 70.6% with an average of 4.3 visits per woman [20]. The prevalence of low birth weight (birth weight of less than 2500 g) was 14.2 and 11.9% in the Ejisu-Juaben municipality and Sekyere-East district respectively. Malaria was reported as the topmost reason for outpatient attendance in both areas [19, 20].

All pregnant women of all parities who were visiting the ANC clinics in the two areas for the first time for their current pregnancy were invited to be part of the study according to the eligibility criteria found in Box 1.

### Box 1 Eligibility criteria

**Table:** 

Inclusion criteria	Exclusion criteria
First time ANC clinic visit for current pregnancy Pregnancy up to 32 weeks gestation Hb ≥ 70 g/l Willing to participate and give her consent	Known history of sickle cell disease, glucose-6-phosphate dehydrogenase deficiency, HIV/AIDS or tuberculosis Presence of significant illness at time of screening, including severe anaemia

### Study design

**Fig. 3:**
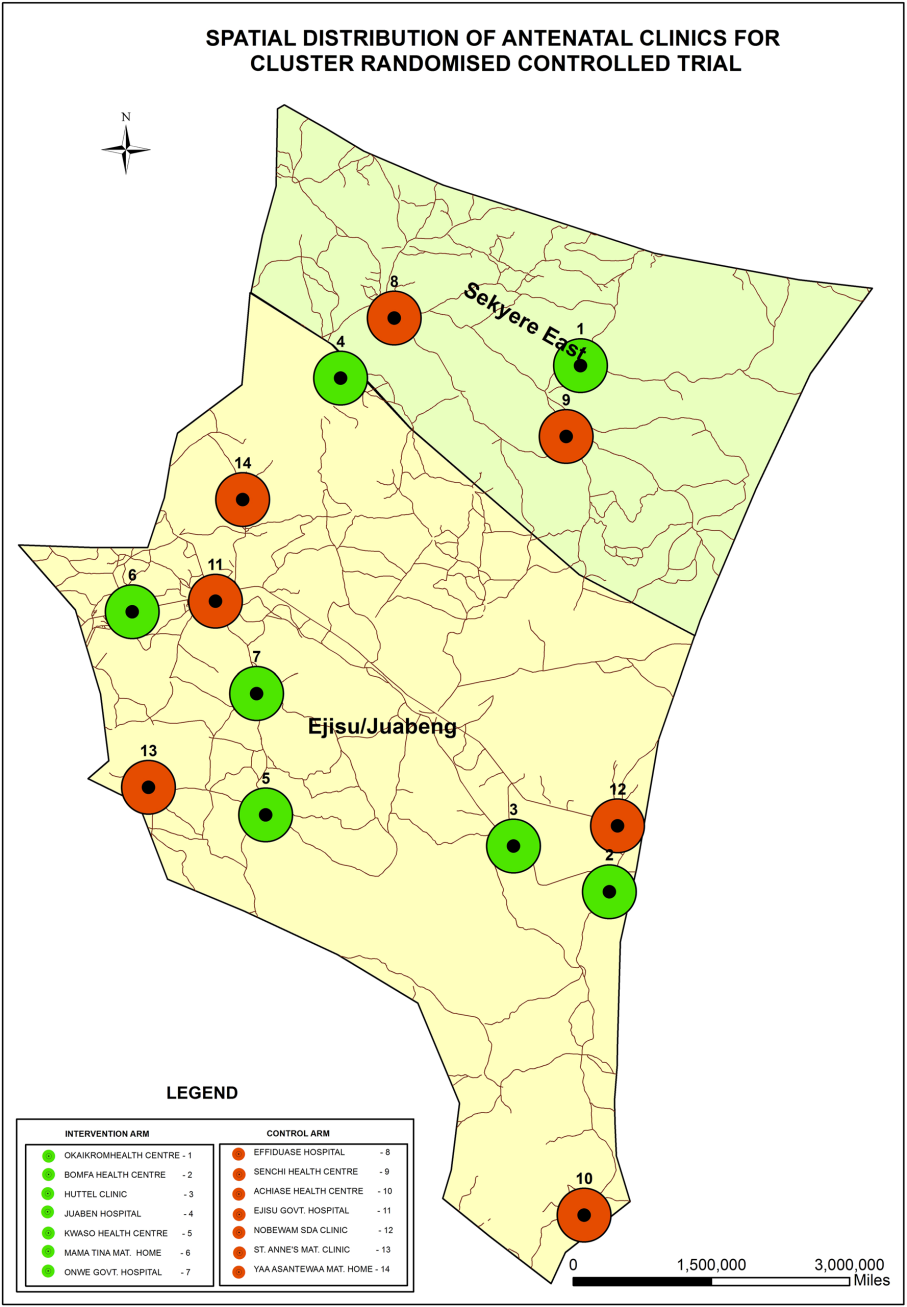
Spatial distribution of antenatal clinics for cluster randomized controlled trial

This was a cluster randomized controlled trial with the ANC clinic being the unit of randomization. Eighteen eligible ANC clinics in the two districts were mapped—using the Global Positioning System to determine their spatial locations and distances between them. An ANC clinic was eligible if registered 10 or more new pregnant women in a month, offered current routine antenatal care services and maternity services. A priori, it was decided that chosen clinics needed to be at least 1.5 km apart so as to reduce the risk of pregnant women crossing over from one ANC clinic to the other during the trial. Based on this, fourteen out of the 18 ANC clinics were finally selected and randomized into intervention and control clinics (Fig. 3) and stratified by the number of registrants (newly booked ANC attendants) per month (large being 20 or more and small being less than 20). A statistician who was independent of the trial performed the randomization using Stata 11 software (StataCorp, College Station, Texas). Each clinic was assigned a unique two-digit code number, which was used to help identify the pregnant women as well and indicated whether they were in the intervention or the control arm of the study. The randomization of the ANC clinics into either the intervention or control arm was not disclosed to the clinic heads and staff during the period of the trial. Table 1 summarizes the features of ANC clinics that were included in the trial.

**Table 1:** Features of ANC clinics included in trial by arm

Feature	Number of ANC clinics	
	Intervention	Control
Location		
Semi-urban	3	4
Rural	4	3
Type		
Hospital	2	2
Health centre	3	3
Community clinic	1	1
Maternity home	1	1
Head of clinic		
Midwife	6	7
Physician assistant	1	0
Routine ANC services offered	7	7
Delivery services offered	7	7

### Sample size

A total of 93 women per ANC clinic was deemed sufficient to detect a 30% decrease in the prevalence of anaemia from 45% [3, 21], with a power of 80 at 95% confidence interval and assuming a co-efficient of variation of 0.15 and an estimated 20% loss to follow up using the formula by Hayes and Bennet [22]. The choice to use anaemia prevalence over parasitaemia prevalence for sample size estimation was informed by

- Reduction in anaemia being the final biological outcome along the pathway of interest in this intervention and
- Anaemia prevalence gives a reasonable number of ANC clinics and pregnant women to be recruited within the number of ANC clinics available in the study area as well as time and budgetary allocation for the trial.

### The intervention

Table 2 summarizes routine recommended ANC services offered in ANC clinics in the study area. These services were carried out in the control and intervention ANC clinics. In addition in the intervention clinics, pregnant women were encouraged to actively participate in anaemia and malaria control aspects of antenatal care using the rapid diagnostic test (RDT), Haemoglobin Colour Scale (HCS) and a pictorial guide (Additional file 1) for malaria and anaemia in pregnancy in the intervention arm. The pictorial guide consists of a series of sketched pictures that depict the causes, signs and symptoms, effects and prevention of malaria and anaemia in pregnancy. It was developed purposely for this study with the help of antenatal care staff in an adjacent district to the study areas to help provide basic and simple health education on malaria and anaemia in pregnancy to the women. The First Response Malaria HRP-2 RDT and the WHO HCS were used in this trial.

**Table 2:** Recommended components of routine antenatal care [34]

History taking	Medical and obstetric history
Physical examination	Blood pressure, weight, height, temperature measurements
Obstetric examination	Symphisio-fundal height measurement, foetal viability
Laboratory tests	Haemoglobin concentration, malaria parasite, Routine urine and stool examination, blood group, sickling, VDRL, HIV
Treatments and immunizations	SP-IPTp, Tetanus toxoid, ACT for malaria, folic acid and iron supplementation, de-worming
Health education and recommendations	ITN use and malaria prevention, dietary, scheduled ANC visit, birth preparedness, danger signs, adherence to medicines, family planning, new born care, post-natal care

The intervention was implemented as described below:

1. During consultation, ANC staff conducted the RDT and HCS tests with the pregnant women using a single finger-tip prick for blood.
2. The pregnant women were invited to see the tests being conducted and the results were interpreted with them.
3. The pregnant women were then encouraged to ask questions concerning the tests and the results.
4. ANC staff used the pictorial guide to provide more information about malaria and anaemia in pregnancy by asking the pregnant women to look at a series of pictures in the guide and give meaning to them.
5. ANC staff then gave health advice and treatments to the pregnant women based on the test results. Health advice included the use of ITN, adhering to iron and folic acid supplementation and anti-malarial treatment, healthy eating and regular ANC clinic attendance.

ANC staff members in the intervention clinics were trained in a 1-day workshop to use the RDT, HCS and pictorial guide to facilitate pregnant women’s active participation in their antenatal care. All pregnant women that attended ANC in the intervention clinics had the intervention administered even if they were not eligible according to the eligibility criteria (Box 1) to be enrolled in the trial. Active participation in their ANC occurred at each visit of the pregnant women until delivery.

### Data collection for assessment of intervention effect

The following trial procedures were undertaken for women in both arms. Enrolment of pregnant women occurred simultaneously in all 14 ANC clinics between September 2012 and December 2013 while follow up was till the end of April 2014.

#### Screening, enrolment and baseline data

1. Pregnant women were screened by trained research assistants using the eligibility criteria (Box 1) for enrolment into the study.
2. Enrolled pregnant women had their socio-demographic, obstetric and medical histories and ITN use recorded.
3. The pregnant women were then allowed to go through their antenatal care.
4. After their antenatal care, blood samples were taken for the measurement of Hb using 301 Hemocue™ analysers (HemoCue, Angelhom, Sweden) and blood slides were done for malaria parasites determination later using microscopy.
5. IPTp administered was recorded.

#### Follow-up

1. Follow-up was at 4–8 weeks after enrolment and prior to delivery at 36–40 weeks gestation as the women visited the ANC clinic.
2. At these follow-up visits, blood samples for Hb and malaria parasite determination were taken again after they had gone through their ANC.
3. At 36–40 weeks gestation, pregnant women were administered questionnaires to assess their level of adherence to iron and folic acid supplementation and their knowledge about malaria and anaemia in pregnancy. Adherence to iron and folic acid supplementation was measured as a proxy for adherence to ANC recommendations or health advice for malaria and anaemia using a visual analogue scale (Additional file 2).
4. At delivery, the birth weight was recorded. Antenatal and maternity registers were checked regularly for women who had reported any adverse outcome of their pregnancies and were recorded throughout the study period.

Additional information on the implementation fidelity of the intervention was collected to help with the interpretation of the trial results. Non-participatory observations of ANC sessions and exit interviews with pregnant women were conducted in the 9th month of the trial. A trained independent observer used a 10-item checklist (Additional file 3) to observe whether the RDT, HCS and pictorial guide were being used accordingly for the active participation of the pregnant women.

Short exit interviews were also conducted with up to 5 pregnant women per ANC clinic on the same day of observations to ascertain the processes they had been through during their ANC according to a 12-item checklist (Additional file 4). The women were shown the RDT, HCS and pictorial guide and asked to confirm or not whether they were used during their ANC for their active participation.

### Data handling and analysis

Data were collected using a pre-designed case report form for each pregnant woman. All data were double entered using Microsoft Access 2007 computer software and discrepancies corrected by manual crosschecking with the case report forms. After data were cleaned, they were analysed using Stata version 13 (StataCorp, College Station, Texas).

Descriptive statistics was used to describe the pregnant women at the individual and ANC clinic levels and any imbalances in covariates and potential confounders identified. The intervention effect was analysed by a modified per protocol analysis. This meant using data available for all women who had successfully been followed up at each pre-specified time point regardless of whether the woman had all measurements recorded for all the time points or not. Assessment of the intervention effect was done at the ANC clinic level as follows: (1) Estimating risks of the endpoints (malaria parasitaemia and anaemia prevalence, low birth weight and sub-optimal pregnancy outcomes-abortions, intrauterine deaths and still births) for each antenatal clinic. (2) Finding the mean risk per arm. (3) Comparing the means using the student T test per arm as risk ratios. All analyses were adjusted for baseline parasitaemia and anaemia, gestation at enrolment, maternal age, educational level, ITN use, parity and IPTp received.

Data for knowledge of pregnant women about malaria and anaemia in pregnancy, adherence to iron and folate supplementation and intervention implementation fidelity were also analysed according to Additional files 5, 6 and 7 and are reported in Tables 7 and 8, respectively.

## Results

### Participant flow throughout the study

**Fig. 4:**
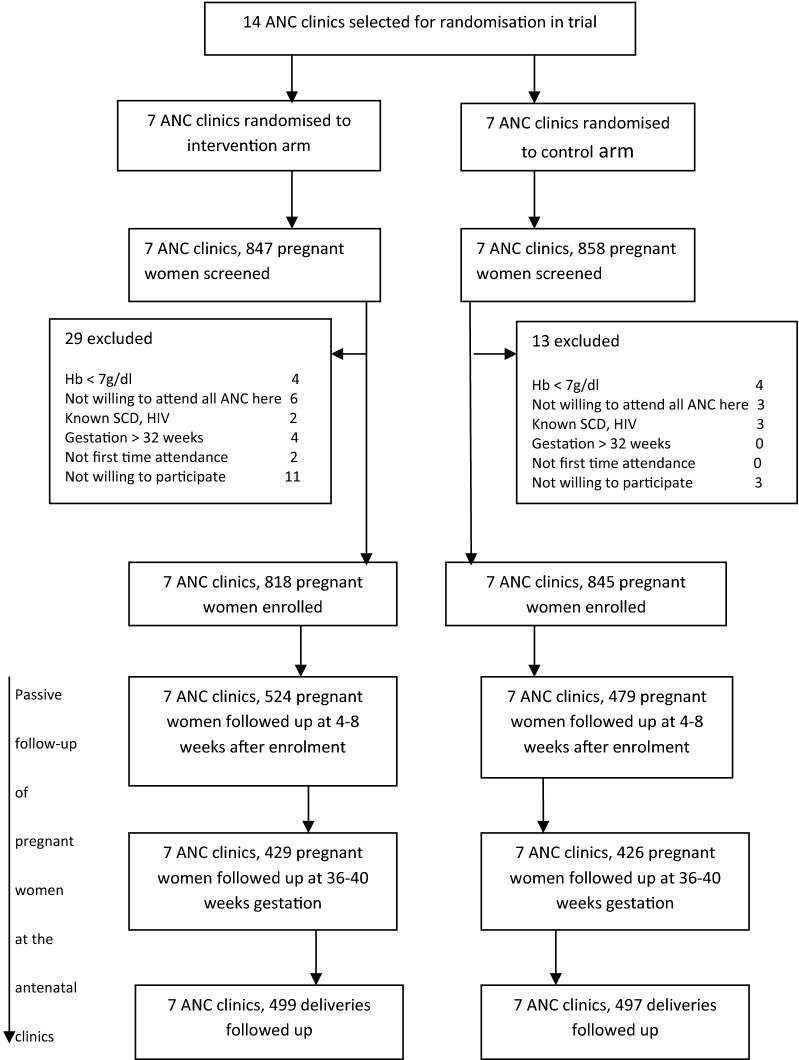
CONSORT diagram showing the flow of participants throughout the trial

Figure 4 and Table 3 show the participant flow throughout the study and the number of women enrolled per ANC clinic respectively. At 4–8 weeks after enrolment, a higher number, 524 out of 818 (64.1%) were followed up in the intervention arm compared with 479 out of 845 (56.7%) in the control arm. Slightly higher numbers of women were followed up in the intervention compared to the control arm prior to delivery and at delivery: 429 (52.4%) in intervention compared to 426 (50.4%) in the control arm prior to delivery and 499 (61%) in the intervention compared to 497 (60.8%) in the control arm.

**Table 3:** Number of pregnant women enrolled per ANC clinic, by study arm

Study arm			
Control		Intervention	
ANC clinic code	Number of women enrolled	ANC clinic code	Number of women enrolled
Achiase	121	Kwaso	120
Nobewam	123	Onwe	122
Senchi	122	Mama Tina	118
Donyinah	119	Okaikrom	97
Yaa Asantewaa	120	Juaben	120
Effiduase	120	Huttel	121
Ejisu	120	Bomfa	120
Total	845		818
Cluster size			
Mean (range)	120.7 (119–123)		116.9 (97–122)

### Characteristics of women at recruitment

Tables 4 and 5 summarize the baseline characteristics of the pregnant women at individual and ANC clinic levels respectively. The mean age of the women was 26.4 years with approximately half of the women in the 20–29 year range (53.7%). Almost 70.0% had given birth to at least one child and more than 50.0% had three or more children. Most of the women attended their first ANC clinic in the first and second trimesters; those attending in the third trimester accounting for less than 13.0%. Overall, 47.2% of the women were anaemic (Hb < 11.0 g/dl) while 10.7% had malaria parasitaemia. A total of 74.6% claimed ownership of bed nets but only 48.8% had slept under it the night before their enrolment Baseline imbalances existed between the two arms with regards to the women’s gravidity, parity, ITN ownership and use. Women in the control arm had higher gravidity, parity, ITN ownership and use compared to those in the intervention group. The mean Hb, malaria parasitaemic densities, age and gestational age at enrolment were however similar in both arms.

**Table 4:** Baseline characteristics of study participants at individual level

Characteristics	Control (N = 845)		Intervention (N = 818)		Total	
	n	%	n	%	n	%
Parity						
0	295	34.95	215	26.28	510	30.69
1	195	23.10	191	23.35	386	23.23
≥ 2	354	41.94	412	50.37	766	46.09
Gravidity						
1	238	28.20	176	21.52	414	24.91
2	189	22.39	182	22.25	371	22.32
≥ 3	417	49.41	460	56.23	877	52.77
Gestation at first ANC visit						
First	324	38.57	260	31.86	584	35.27
Second	414	49.29	451	55.27	865	52.23
Third	102	12.14	105	12.87	207	12.5
Mean (SD) gestational age	15.93	7.12	17.08	6.74		
Age group						
Under 20	125	14.81	134	16.38	259	15.58
20–29	470	55.69	423	51.71	893	53.73
30 and above	249	29.50	261	31.91	510	30.69
Mean age (SD)	26.29	6.37	26.44	6.64		
Educational level						
None	87	10.32	127	15.54	214	12.89
Basic	617	73.19	611	74.79	1228	73.98
Post basic	139	16.49	79	9.67	218	13.13
ITN ownership						
Yes	574	68.09	665	81.3	1239	74.59
ITN use						
Yes	341	40.45	470	57.46	811	48.83
IPT-SP served						
Yes	239	28.35	294	36.16	533	32.19
Anaemia						
Yes	381	45.14	403	49.27	784	47.17
Mean haemoglobin (SD) (g/dl)	11.03	(1.46)	10.99	(1.44)		
Parasitaemia						
Yes	64	7.69	111	13.70	175	10.66
GMPD (95% CI)	653.45 (481.56–886.68)		442.76 (332.01–590.46)

**Table 5:** Baseline characteristics of study participants at ANC clinic level

	Control	Intervention	*p* value
Number of clusters (ANC clinics)	7	7	
Hb			
Mean of ANC clinic summaries	11.03	10.99	0.83
Standard deviation	0.38	0.33	
Median	11.06	11.01	
Range	10.55–11.47	10.61–11.63	
Age			
Mean of ANC clinic summaries	26.29	26.40	0.83
Standard deviation	0.92	0.98	
Median	25.88	26.11	
Range	25.22–27.54	25.07–27.89	
Gestational age at enrolment (weeks)			
Mean of ANC clinic summaries	15.92	17.12	0.26
Standard deviation	1.95	1.86	
Median	16.01	18.03	
Range	13.07–18.95	14.42–19.18	
Parity			
Mean of ANC clinic summaries	1.60	1.95	0.03
Standard deviation	0.24	0.28	
Median	1.54	1.84	
Range	1.28–2.05	1.66–2.50	
Gravidity			
Mean of ANC clinic summaries	2.93	3.24	0.03
Standard deviation	0.21	0.27	
Median	3.00	3.23	
Range	2.61–3.16	2.92–3.80	
Parasite density			
Mean of ANC clinic summaries	1202.95	1625.57	0.40
Standard deviation	601.19	1138.95	
Median	989.33	1300.00	
Range	543.33–2014	559.20–4087	
Anaemia			
Mean of ANC clinic proportions	45.08	49.23	0.50
95% CI	(33.20–56.96)	(40.51–57.96)	
Felt unwell			
Mean of ANC clinic proportions	54.09	66.47	0.28
95% CI	(31.76–76.42)	(52.02–80.92)	
ITN ownership			
Mean of ANC clinic proportions	67.90	81.55	0.004
95% CI	(62.44–73.36)	(73.98–89.12)	
ITN use			
Mean of ANC clinic proportions	40.28	58.03	0.03
95% CI	(31.58–48.97)	(42.09–73.96)	
Parasitaemia			
Mean of ANC clinic proportions	9.14	15.86	
95% CI	(2.93–15.36)	(8.57–23.15)	0.11

### Study outcomes

The intervention effect is summarized in Table 6. Generally, malaria parasitaemia prevalence decreased in both study arms by the end of pregnancy to less than 6.0% compared to the baseline value of 10.7%. Anaemia prevalence however saw an increase to slightly over 50.0% in the control arm while that in the intervention arm virtually saw no change over the baseline value of 47.2%.

**Table 6:** Intervention effect on anaemia, malaria parasitaemia, low birth weight and sub optimal pregnancy outcomes

	Clusters	Risk (%)	95% confidence interval	Unadjusted risk ratio	95% confidence interval	p-value	Adjusted risk ratio^a^	95% confidence interval	p-value
Anaemia at 4–8 weeks after enrolment									
Control	7	48.39	33.33–70.00						
Intervention	7	48.04	36.74–62.63	0.99	0.73–1.34	0.96	0.97	0.78–1.22	0.79
Anaemia prior to delivery									
Control	7	50.25	22.73–78.95						
Intervention	7	47.40	20.41–65.38	0.94	0.60–1.49	0.78	0.92	0.63–1.34	0.62
Malaria at 4–8 weeks after enrolment									
Control	7	6.43	1.67–12.82						
Intervention	7	6.73	2.60–11.50	1.05	0.58–1.89	0.86	1.17	0.68–2.04	0.51
Malaria prior to delivery									
Control	7	5.91	0.00–15.00						
Intervention	7	4.34	0.00–7.690	0.73	0.24–2.26	0.59	0.83	0.27–2.57	0.73
Low birth weight									
Control	7	13.18	5.41–31.48						
Intervention	7	10.81	2.44–19.74	0.82	0.38–1.78	0.59	0.93	0.44–1.97	0.84
Suboptimal pregnancy outcome									
Control	7	8.35	0.00–39.22						
Intervention	7	6.32	0.00–10.53	0.76	0.16–3.48	0.72	0.77	0.17–3.52	0.73

^a^Risk ratios adjusted for baseline parasitaemia, anaemia, gestation at enrolment, age, educational level, ITN use, IPT-SP served and parity

The risk of maternal anaemia did not vary significantly between the two groups during pregnancy. At 4–8 weeks after enrolment, the risk of anaemia was reduced marginally by 3.0% in the intervention group compared to the control [adjusted risk ratio (ARR) 0.97; 95% CI 0.78–1.22] and by 8.0% prior to delivery (ARR 0.92; 95% CI 0.63–1.64). Similarly, there was no significant difference between the groups with regards to the risk of malaria parasitaemia during pregnancy. At 4–8 weeks after enrolment, the intervention group appeared to have a higher risk of parasitaemia of 17.0% (ARR 1.17; 95% CI 0.68–2.04) compared to the control group but prior to delivery, the risk of parasitaemia had reduced by 17.0% in the intervention arm compared to the control group (ARR 0.83; 95% CI 0.27–2.57). The risk of low birth weight and sub-optimal pregnancy outcomes did not vary significantly between the study groups although low birth weight reduced by 7.0% (ARR 0.93; 95% CI 0.44–1.97) in the intervention group compared to the control and the risk of sub-optimal pregnancies reduced by 23.0% (ARR 0.77; 95% CI 0.17–3.52).

## Discussion

This was a pragmatic cluster randomized controlled trial designed to investigate the effectiveness of pregnant women’s active participation in their antenatal anaemia and malaria point-of-care testing in improving pregnancy outcomes. The study showed a potential of improving maternal and pregnancy outcomes since the risk of malaria parasitaemia, anaemia, low birth weight and sub-optimal pregnancies appeared reduced in the intervention arm compared to the control arm by the end of pregnancy although not significantly.

A limitation identified in this study is more than 30% loss to follow-up at each time point in both arms (Fig. 4). Also, an assumption of a 30% intervention effect based on documented effects of patient participation in previous studies [23–25] may not have been applicable to our study and may have also contributed to reduced power to robustly detect significant benefit.

The nature of the trial, being pragmatic, did not call for active follow up into the communities and this may have contributed to a reduced power to detect significant intervention effect. Pregnant women’s ANC seeking behaviour may be influenced by numerous factors including the educational level of the woman and her husband, distance to the clinic from their homes, direct (payment for some services at the clinic) and indirect costs (transportation, food to eat and time spent to seek care), complacency among elder women, and women’s previous experiences with ANC and the staff [26–28]. Although these factors were not explored in this current study, they may have contributed to the loss to follow up. In order to achieve the maximum rate of data capture as the women attended their subsequent ANC visits, research assistants were stationed at the clinics every day of the week to be able to identify participants for follow up at the clinics. It is thus likely that this may be a true reflection of what pertains in the study area. However understanding what factors affect ANC seeking behaviour in subsequent visits in the study area may be necessary to help improve ANC re-attendance.

The intervention may have been responsible for the higher number of women re-attending the 4–8 weeks after recruitment follow-up visit in the intervention arm compared to the control arm (Fig. 4). Women in the intervention arm may have been curious to see whether there had been changes in their Hb and malaria parasitaemia levels after their first visit, hence motivated to visit the clinic at their scheduled date, accounting for the higher numbers. This may also have accounted for the higher risk of parasitaemia detected in the intervention arm compared to the control arm at this time point as a higher number of women were tested compared to the control arm. But this motivation was not enough to sustain attendance during pregnancy with, both arms recording similar reduced attendance at term and at delivery. Other factors like the age, parity, educational status, socio- economic situation and empowerment of the women [27, 29, 30] may be of equal importance in determining re-attendance to antenatal care.

The risk of parasitaemia recorded at term pregnancy in both arms was lower (Table 6) than has previously been reported [3, 4, 21]. The deployment of RDT to all ANC clinics by the Ghana Health Service during the time of the trial meant that RDT became available in the control ANC clinics as well. This may have reduced the effect of the intervention as it is possible that some ANC staff may have allowed the pregnant women in the control group to see their test results (as reported in a qualitative study yet to be published elsewhere). None the less, more widespread use of RDT may have helped identify more women with parasitaemia for treatment during pregnancy hence reducing their risk of malaria at term. The HCS and pictorial guides were at the intervention clinics only so these may have accounted for the slightly lower but not significant risk of anaemia observed in the intervention arm compared to the control arm.

Improved adherence of pregnant women to ANC treatments and recommendations was anticipated as a benefit of active participation of pregnant women in their care; however no significant difference was measured between intervention and control groups (Table 7). Patients’ adherence is a complex health behaviour, and has been found to be affected by a complex interplay of other factors including attitudes, beliefs and group norms; culture; psychological state, the patient’s involvement and participatory decision making level, type of illness and co-morbidity, socio-economic level, use of alternative medicine and stakes of proposed outcome [13, 17]. Understanding what factors influenced these pregnant women’s adherence to ANC recommendations and treatments might have helped explain the results in the context of complex inter-relations between patient adherence, improved health outcomes and intervention implementation. Health literacy, another factor that affects patient adherence [13] was slightly but not significantly increased in the intervention group compared to the control group. This was evidenced by the higher scores of adequate knowledge about malaria and anaemia in pregnancy (Table 7) in the intervention group compared with the control group however this alone may not have been enough to positively influence the pregnant woman’s level of adherence.

**Table 7:** Comparison of mean proportions of women’s knowledge on malaria, anaemia and adherence by arm

	Study arm	Mean proportion	Standard deviation	95% confidence interval	p-value
Adequate knowledge on anaemia	Control	31.28	23.37	9.67–52.89	
	Intervention	43.81	35.07	11.37–76.24	0.4468
Adequate knowledge on malaria	Control	40.59	27.80	14.87–66.30	
	Intervention	54.26	30.83	25.75–82.78	0.4005
Adequate adherence to health advice	Control	34.20	23.31	12.63–55.76	
	Intervention	35.86	17.54	19.64–52.08	0.8822

Although patient adherence to health interventions is important for health intervention effectiveness, health service factors including accessibility to healthcare, availability of diagnostics and health provider compliance with intervention implementation [6] are necessary for intervention effectiveness too. In this study, it was assumed that financial accessibility to antenatal care was assured by the free maternal health care and delivery policy that was underway in the country at time of the study [31] and geographical accessibility by the design of the study. There was also constant supply of the point-of-care tests (diagnostics) needed throughout the study since these were provided by the project. Health provider compliance with intervention implementation, measured during the study as implementation fidelity was however found to be below expectation (Table 8). ANC staff may have adopted the active participation concept with enthusiasm at the beginning of the trial but this was not sustained as evidenced by the lower than expected implementation fidelity. Health workers’ unwillingness to delegate power, their perceived lack of time, the type of health situation the health care worker is dealing with, their individual characteristics including their personal beliefs, perceptions and knowledge of the intervention, their specialization level, and their age and sex are known to influence their level of compliance [17, 32]. Although these factors were not explored in this study, some of them may have contributed to the sub-optimal implementation and hence reduced intervention effectiveness.

**Table 8:** Implementation fidelity-Intervention implementation activities against expected activities (percentage of agreement-POA) of intervention clinics

ANC clinic code	Pregnant women’s report				Observations		
	Interviewee code	Activities conducted	Activities not conducted	POA	Activities observed	Activities not observed	POA
Kwaso	K1	12	0	73.00	7	3	70.0
	K2	10	2				
	K3	7	5				
Onwe	O1	2	10	17.0	2	8	20.0
	O2	2	10				
	O3	2	10				
Mama Tina	M1	4	8	33.0	3	7	30.0
	M2	4	8				
	M3	0	12				
Okaikrom	Ok1	2	10	17.0	2	8	20.0
Juaben	J1	7	5	58.0	7	3	70.0
	J2	8	4				
	J3	4	8				
Huttel	H1	7	5	38.0	4	6	40.0
	H2	3	9				
	H3	4	8				
	H4	4	8				
Bomfa	B1	4	8	70.0	8	2	80.0
	B2	3	9				
	B3	11	1				
	B4	12	0				
	B5	12	0				
Average POA				43.7			47.1

## Conclusion

This study has demonstrated that encouraging pregnant women to see and be involved with the interpretation of their point-of-care test results for malaria and anaemia has the potential to improve maternal and pregnancy outcomes albeit not statistically significant. It appears there is a rather complex inter-relation between active patient participation, patient adherence, intervention implementation and expected health outcomes. Exploring what factors influence health worker compliance to health intervention implementation and patient adherence to health interventions within this context will contribute to understanding and improving intervention effectiveness and hence patient health outcomes.

## Additional files

**Additional file 1: Figure S1.** A pictorial guide to the common symptoms, effects and prevention of malaria and anaemia in pregnancy (https://drive.google.com/file/d/0B_7q9tDk78T8ZlRoVW5kNkdHd00/view?usp=sharing).

**Additional file 2: Figure S2.** The visual analogue scale (VAS).

**Additional file 3: Table S1.** Checklist for the observation of the implementation of the ‘client participation’ intervention.

**Additional file 4: Table S2.** Checklist for the pregnant women’s report of the ‘client participation’ intervention.

**Additional file 5: Box S1.** Assessment of level of knowledge about malaria and anaemia in pregnancy.

**Additional file 6: Box S2.** Assessment of level of adherence.

**Additional file 7: Box S3.** Measurement of implementation fidelity.

## Abbreviations

ANC: antenatal care
CI: confidence interval
DHMT: District Health Management Team
Hb: haemoglobin concentration
HIV/AIDS: human immune-deficiency virus/acquired immune deficiency syndrome
IPTp: intermittent preventive treatment of malaria in pregnancy
ITN: insecticide-treated bed net
MHMT: Municipal Health Management Team
SP: Sulfadoxine–pyrimethamine
