# Supplementary material for: Effectiveness of pregnant women’s active participation in their antenatal care for the control of malaria and anaemia in pregnancy in Ghana: a cluster randomized controlled trial
Source: Malar J. 2018 Jun 19;17:238. doi: 10.1186/s12936-018-2387-1 (PMC6009977; doi:10.1186/s12936-018-2387-1)
Supplement: Supplementary file 1 — Additional file 1: Figure S1. A pictorial guide to the common symptoms, effects and prevention of malaria and anaemia in pregnancy (https://drive.google.com/file/d/0B_7q9tDk78T8ZlRoVW5kNkdHd00/view?usp=sharing). [file 12936_2018_2387_MOESM1_ESM.docx]

Figure S1: A pictorial guide to the common symptoms, effects and prevention of malaria and anaemia in pregnancy *(https://drive.google.com/file/d/0B_7q9tDk78T8ZlRoVW5kNkdHd00/view?usp=sharing)*
