# Supplementary material for: Effectiveness of pregnant women’s active participation in their antenatal care for the control of malaria and anaemia in pregnancy in Ghana: a cluster randomized controlled trial
Source: Malar J. 2018 Jun 19;17:238. doi: 10.1186/s12936-018-2387-1 (PMC6009977; doi:10.1186/s12936-018-2387-1)
Supplement: Supplementary file 2 — Additional file 2: Figure S2. The visual analogue scale (VAS). [file 12936_2018_2387_MOESM2_ESM.docx]

**0 50% 100**

|  | | | | |  | | | | |
| --- | --- | --- | --- | --- | --- | --- | --- | --- | --- |
|  |  |  |  |  |  |  |  |  |  |
|  |  |  |  |  |  |  |  |  |  |

Figure S2: The visual analogue scale (VAS)
