# Supplementary material for: Effectiveness of pregnant women’s active participation in their antenatal care for the control of malaria and anaemia in pregnancy in Ghana: a cluster randomized controlled trial
Source: Malar J. 2018 Jun 19;17:238. doi: 10.1186/s12936-018-2387-1 (PMC6009977; doi:10.1186/s12936-018-2387-1)
Supplement: Supplementary file 3 — Additional file 3: Table S1. Checklist for the observation of the implementation of the ‘client participation’ intervention. [file 12936_2018_2387_MOESM3_ESM.docx]

Table S1: Checklist for the observation of the implementation of the 'client participation' intervention

| Name of Health facility: [____________________________________________________________] |  |  |
| --- | --- | --- |
| Health facility code: [___/___] |  |  |
| Date of visit: [____/_____/_________] |  |  |
| Observer code: [___/___] |  |  |
|  |  |  |
| **Activity Observed** | **Yes** | **No** |
| 1. Group education of pregnant women about malaria and anaemia using the pictorial guide |  |  |
| 1. One on one education of pregnant women about malaria and anaemia using the pictorial guide |  |  |
| 1. ANC staff using the rapid diagnostic test (RDT) to test for malaria in pregnant women |  |  |
| 1. ANC staff using the Haemoglobin colour scale (HCS) to test for anaemia in pregnant women |  |  |
| 1. ANC staff engaging pregnant women in the interpretation of results of the RDT |  |  |
| 1. ANC staff engaging pregnant women in the interpretation of results of the HCS |  |  |
| 1. ANC staff giving recommendations to pregnant women based on the results of RDT |  |  |
| 1. ANC staff giving recommendations to pregnant women based on the results of HCS |  |  |
| 1. ANC staff giving the pregnant women the opportunity to ask questions about antenatal consultation |  |  |
| 1. ANC staff recording results of the HCS and RDT in note book |  |  |
