# Supplementary material for: Effectiveness of pregnant women’s active participation in their antenatal care for the control of malaria and anaemia in pregnancy in Ghana: a cluster randomized controlled trial
Source: Malar J. 2018 Jun 19;17:238. doi: 10.1186/s12936-018-2387-1 (PMC6009977; doi:10.1186/s12936-018-2387-1)
Supplement: Supplementary file 4 — Additional file 4: Table S2. Checklist for the pregnant women’s report of the ‘client participation’ intervention. [file 12936_2018_2387_MOESM4_ESM.docx]

Table S2: Checklist for the pregnant women's report of the 'client participation' intervention

| Name of Health facility:[_______________________________________________________________] |  |  |
| --- | --- | --- |
| Health facility code: [___/___] |  |  |
| Date of visit: [____/_____/_________] |  |  |
| Pregnant woman code: |  |  |
| Observer code: |  |  |
| **Activity described by pregnant woman** | **Yes** | **No** |
| 1. Seen the rapid diagnostic test (RDT) before |  |  |
| 1. RDT used to test her blood during ANC session |  |  |
| 1. Saw the results of RDT |  |  |
| 1. Results of RDT interpreted with her |  |  |
| 1. Seen the Haemoglobin colour scale (HCS) before |  |  |
| 1. HCS used to test her blood during ANC session |  |  |
| 1. Saw the results of HCS |  |  |
| 1. Results of HCS interpreted with her |  |  |
| 1. Seen pictorial guide before |  |  |
| 1. Pictorial guide used during health talks and/ or one-on-one |  |  |
| 1. Asked to talk about pictures in the pictorial guide |  |  |
| 1. ANC staff gave health advice based on test results |  |  |
