# Supplementary material for: Effectiveness of pregnant women’s active participation in their antenatal care for the control of malaria and anaemia in pregnancy in Ghana: a cluster randomized controlled trial
Source: Malar J. 2018 Jun 19;17:238. doi: 10.1186/s12936-018-2387-1 (PMC6009977; doi:10.1186/s12936-018-2387-1)
Supplement: Supplementary file 5 — Additional file 5: Box S1. Assessment of level of knowledge about malaria and anaemia in pregnancy. [file 12936_2018_2387_MOESM5_ESM.docx]

| Box S1: Assessment of level of knowledge about malaria and anaemia in pregnancy |
| --- |
| - Knowledge about malaria or anaemia was constructed from the responses to five questions about the causes, signs and symptoms, effects of the diseases on mother and on baby and prevention of the disease. |
| - Each question had at least 3 three possible correct answers. |
| - For each question, the woman was given the opportunity to mention as many answers as possible and these were ticked. |
| - If the woman was able to mention at least one correct answer for each of the five questions, she was assessed to have adequate knowledge. |
| - If she was not able to answer at least one correct answer for any of the 5 questions, then she was assessed as having poor knowledge. |
