# Supplementary material for: Effectiveness of pregnant women’s active participation in their antenatal care for the control of malaria and anaemia in pregnancy in Ghana: a cluster randomized controlled trial
Source: Malar J. 2018 Jun 19;17:238. doi: 10.1186/s12936-018-2387-1 (PMC6009977; doi:10.1186/s12936-018-2387-1)
Supplement: Supplementary file 6 — Additional file 6: Box S2. Assessment of level of adherence. [file 12936_2018_2387_MOESM6_ESM.docx]

| Box S2: Assessment of level of adherence |
| --- |
| - Self-reported adherence to iron and folate supplementation was measured using the visual analogue scale (VAS) |
| - The VAS is divided into 10% points from 0% to 100% across the scale - The use of the VAS was explained to each pregnant woman before her estimation |
| - Each pregnant woman estimated how much of her supplementation she had taken over a 2 week period against the expected intake |
| - She then pointed to it on the VAS and this was marked |
| - Adequate adherence was assessed as a mark of 60% or higher |
| - Low adherence was assessed as a mark of 50% or lower |
