# Supplementary material for: Effectiveness of pregnant women’s active participation in their antenatal care for the control of malaria and anaemia in pregnancy in Ghana: a cluster randomized controlled trial
Source: Malar J. 2018 Jun 19;17:238. doi: 10.1186/s12936-018-2387-1 (PMC6009977; doi:10.1186/s12936-018-2387-1)
Supplement: Supplementary file 7 — Additional file 7: Box S3. Measurement of implementation fidelity. [file 12936_2018_2387_MOESM7_ESM.docx]

| Box S3: Measurement of implementation fidelity |
| --- |
| - Percentage of agreement (POA) between a)observed and b)reported activities and expected implementation activities was computed as follows: |
| 1. Total number of activities observed per ANC clinic or reported by pregnant woman = x |
| 2. Total number of activities on check list = y |
| 3. POA = (x/y) X 100 |
| - Thus for observed activities per ANC clinic: |
| POA = (x/10) X 100 |
|  |
| - And for pregnant women's reported activities |
| POA= (x/12) X 100 |
| - An average POA was computed for pregnant women's report for each clinic by summing up the individual women’s POA and dividing by the total number of women interviewed for that clinic. |
